# Analysis of endophyte diversity of two *Gentiana* plants species and the association with secondary metabolite

**DOI:** 10.1186/s12866-022-02510-4

**Published:** 2022-04-07

**Authors:** Qin Zheng Hou, Da Wei Chen, Yu Pei Wang, Nurbiye Ehmet, Jing Ma, Kun Sun

**Affiliations:** 1 College of Life Sciences, Northwest Normal University, Lanzhou, 730070 Gansu China; 2 Gansu Provincial Maternity and Child-Care Hospital, Lanzhou, 730000 Gansu China

**Keywords:** *G. officinalis*, *G. Siphonantha*, Endophytes, Diversity, Correlation analysis, Ecological function

## Abstract

**Background:**

The influence of external environmental factors on secondary metabolites of medicinal plants has always been studied. However, little is known about the relationships between endophytes and host metabolites, especially the relationship differences between different plant species. Thus, we used high-throughput sequencing methods to compare endophyte diversity from roots of two closely related species, *Gentiana officinalis* and *G. siphonantha*, from the same production area, and analyze the association with four secondary metabolites (Gentiopicroside, Loganic acid, Swertiamarine and Sweroside).

**Results:**

The fungal and bacteria communities’ richness and diversity of *G. siphonantha* was higher than *G. officinalis*. Ascomycota and Proteobacteria were dominant fungal and bacterial phylum of the two closely related species. At the genus level, *Tetracladium* and *Cadophora* were dominant fungal genus in *G. officinalis* and *G. siphonantha* samples, respectively. While *Pseudomonas* was dominant bacterial genus in two closely related species, with relative abundances were 8.29 and 8.05%, respectively. Spearman analysis showed that the content of loganic acid was significantly positively correlated with endophytic fungi, the content of gentiopicroside, swertiamarine and sweroside were significantly positively correlated with endophytic bacteria in the two related species*.* PICRUSt and FUNGuild predictive analysis indicated that metabolism and saprotroph was primary function of endophytic bacteria and fungi in the two related species*.*

**Conclusion:**

Our results will expand the knowledge on relationships of plant-microbe interactions and offer pivotal information to reveal the role of endophytes in the production of *Gentiana* plant and its important secondary metabolite.

**Supplementary Information:**

The online version contains supplementary material available at 10.1186/s12866-022-02510-4.

## Introduction

Endophytes existed the internal tissues of plant, but it can not cause any disease symptoms, which played a vital role in plant growth, development, tolerance of abiotic or biotic stress and accumulation of host secondary metabolites [1, 2]. Chen et al. [3] found that the five secondary metabolites content of *Rheum palmatum* were positively correlated with the fungal endophyte. Song et al. [4] reported that *Bacillus altitudinis* was isolated from *Panax ginseng,* which can improve ginsenoside accumulation. Gao et al. [5] reported that endophytic *Paenibacillus polymyxa* can promote *P. ginseng* growth, improved ginsenoside content, and decreased plant disease. Many studies proved that the endophytes composition were affected by plant species, parts, and growth stage [6]. It was important to analyze the diversity and composition of endophyte in plant parts for plant growth promotion and biotransformation [7].

Sect. *Cruciata* (Qinjiao in Chinese) belong to the *Gentiana* genus [8], which were widely distributed in the northern hemisphere [9]. In China, it has been used as herbalism about 2000 years, and was normally one of an ingredient in some traditional formulae. The main active ingredients of the Qinjiao included gentiopicroside, loganic acid, swertiamarine and sweroside. The biological and pharmacological effects of Qinjiao was reported, such as anti- inflammatory, antifungal, antihistamine and antihepatotoxic activities, which was now officially recorded in the National Pharmacopoeia of China [10, 11]. In recent years, the wild stock of this species has decreased more rapidly than ever and most natural populations have been destroyed in order to meet the commercial demand for the genuine *Gentiana* species [12]. Therefore, it was important to understand the biology of the crop and find scientific practices to replace the traditional modes of Qinjiao cultivation.

Previous studies have reported that the secondary metabolites of Sect. *Cruciata* plants were different [13]. At present, a lot of research focused that external ecological environment and the gene impact medicinal plants, such as temperature, rainfall, light and so on [14]. However, the effect of internal environment of medicinal plants was seldomly studied. In particular, endophytes and medicinal plants composed microecosystem in the internal environment of medicinal plants, which will help explain the reasons of the quality difference of different *Gentiana* species from a new perspective. Additionaly, species of Sect. *Cruciata* are sister species, and they are common origin because of radiation differentiation of Sect. *Cruciata* [14]*.* Whether these closely related species of Sect. *Cruciata* have different endophytes, and whether different secondary metabolites of Sect. *Cruciata* plants correlated with endophytes? The answers of these questions will much helpful in deeply understanding the relationships between plants and endophytes. However, it is still quitely unknown about this.

Therefore, in this study, two species of Sect. *Cruciata*, *G. officinalis* and *G. siphonantha*, were collected from same production area, and the primary goals of this work were as follows: (1) compare endophytes diversity of *G. officinalis and G. siphonantha.* (2) predict the endophytic bacterial and fungal functions of *G. officinalis* and *G. siphonantha*. (3) analyze the relationship between endophytes and host metabolites. These results may lay a foundation for expanding the knowledge on plant–microbe relationships and boosting Qinjiao quality.

## Materials and methods

### Experimental materials

**Fig. 1:**
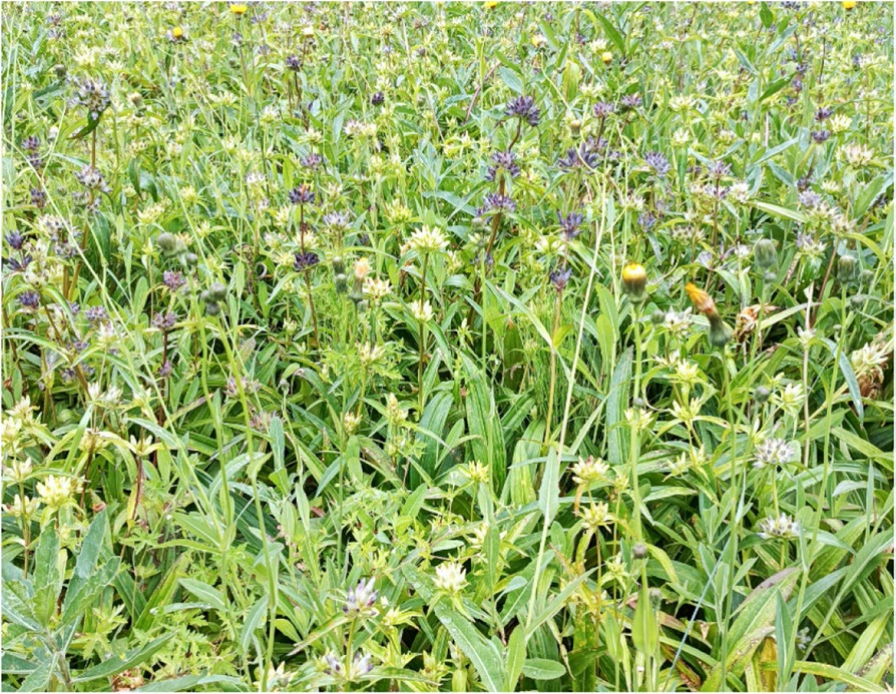
The mixed planting of *Gentiana officinalis* and *G. siphonantha* in the same field

Three-year-old roots of *G. officinalis* and *G. siphonantha* were collected from Tianzhu county, Wuwei city, Gansu Province, China (102°33′34″, 34°58′1″, 2480 m, mean annual precipitation: 408.3 mm, mean annual temperature: 0.3 °C), the both were mixed planting in the same field, internal distance among individuals was kept above 20 m basically (Fig. 1), three biological replicates were selected for uniformity based on size and weight. The samples were separated and washed with running tap water, then rinsed thrice with distilled water. A single sample consisted of 0.5 g of each part from plants as one sample. To sterilize the surface of the plant parts, the root samples were successively immersed in 70% ethanol for 5 min, 2.5% sodium hypochlorite for 1–2 min, and 70% ethanol for 1 min, and then rinsed five times with sterile Millipore water. The last portion of the washing water was inoculated in PDA (potato dextrose agar) at 28 °C for 10 d and NA (nutrient agar) at 37 °C for 3 d to validate sterilization efficiency [3]. All samples were stored at − 80 °C until DNA extraction.

### DNA extraction, polymerase chain reaction (PCR) amplifcation, and sequence processing

The total genomic DNA was extracted from all samples by using the MOBIO Power -Soil® Kit (MOBIO Laboratories, Inc., Carlsbad, CA, USA), according to the manufacturer’s instructions. The DNA extracts were analyzed for their concentration using NanoDrop spectrophotometer (Termo Fisher Scientifc, Model 2000, MA, USA) and stored at − 20 °C for further PCR amplifcation. The PCR assays were performed in 20 μL mixture containing 4 μL of 5× Fast-Pfu buffer, 2 μL of 2.5 mM dNTPs, 0.8 μL of each primer (5 μM), 0.4 μL of FastPfu Polymerase, 10 ng of templateDNA and ddH_2_O. Bacterial 16S gene was amplified with primers 338F (5′-ACTCCTACGGGAGGCAGCA-3′) and 806R (5′-GGACTACHVGGGTWTCTAAT-3′) [3]. Amplification was performed under the following conditions: initial denaturation at 95 °C for 3 min, 30 cycles at 95 °C for 30 s, 52 °C for 30 s, and 72 °C for 45 s, and a final extension at 72 °C for 5 min. The fungal ITS genes were amplifed using the primers ITS1F (5′-CTTGGTCATTTAGAGGAAGTAA-3′) and ITS2R (5′-GCTGCGTTCTTCATCGATGC-3′) [3]. The PCR reactions were conducted using the following program: 3 min of denaturation at 95 °C, 35 cycles of 30 s at 95 °C, 30 s for annealing at 55 °C, and 45 s for elongation at 72 °C, and a final extension at 72 °C for 10 min. The PCR products were analyzed by agarose gel electrophoresis. For each sample, three successful PCR products were pooled and purified using EasyPureTM PCR Clean up / Gel Extraction Kit (Axygen Biosciences, Union City, CA, U.S.) according to manufacturer’s instructions. Purified amplicons were sequenced on an Illumina NovaSeq platform for paired-ends according to the standard protocols [15].

### Metabolites of *Gentiana* plants quantitative analysis

Standard gentiopicroside, loganic acid, Swertiamarine and Sweroside were obtained from Shanghai R&D Center for Standardization of Traditional Chinese Medicines. High-performance liquid chromatography (HPLC)- ultrapure water, analytical-grade methanol and phosphoric acid were purchased from Sangon Biotech, Ltd. (Shanghai, China).

The dried root of each treatment specimen (three replications) was pulverized and sieved through a 300 μm mesh. A total of 1.0 of powder of each sample was precisely weighed and added 20 mL methanol, and treated with ultrasound (30 ~ 40 °C, 250 W, 50kHZ) for 30 min. Filtrate was obtained by filtration of 0.22 μm Millipore filter unit, and 10 μL of sample solution was injected into HPLC for determination.

The samples were analyzed by HPLC (Waters.) using C18 (4.6 × 250 mm, 5.0 μm, Waters E2695, USA) at 30 °C, and the content of metabolites was determined: The mobile phase was methanol (A) - 0.15% phosphoric acid (B). 0–4 min, 25% A; 4–12 min, 25 -33% A; 12–20 min, 33 - 40% A; 20–25 min, 40 - 25% A. The flow rate was 1 mL·min^− 1^. The detection wavelength was 242 nm.

### Data analysis

The data were analyzed by utilizing the QIIME pipeline, as previously performed in methods of Ryan et al. [16]. Fungal and bacterial sequences were trimmed and assigned to each sample based on their barcodes. The UPARSE-OTUref was used to classify OTUs at the species level by searching all sequences against the Silva bacterial 16S database [17]. OTUs were classified at the species level by searching against the UNITE fungal database [18]. Sequences were binned into operational taxonomic units (OTUs) at 97% similarity level by using USEARCH software (http://drive5.com/uparse/) [17]. Rarefaction analysis based on Mothur v.1.21.1 was conducted to reveal the diversity indices, including goods_coverage, Chao 1 and Shannon [19]. Non-Metric Multi-Dimensional Scaling (NMDS) analysis were used to analyze the community differences between different samples based on Bray-Curtis [20]. Metabolic and ecologically relevant functions were annotated by PICRUSt for the 16S rDNA OTU and FUNGuild v1.0 for the ITS OUT [21]. Correlation analysis between metabolites and diversity of endophytes was used Spearman method [19]. The data were analyzed by SPSS16.0 software for variance (one-way, ANOVA) and Duncan’s multiple range test (*P* < 0.05).

## Results

### Surface-sterilization efficiency

The results showed that no colonies were observed in PDA and NA medium after a certain period of cultivation, it reflected that the method of surface-sterilization was effective, and the surface-sterilized samples can be used for subsequent tests.

### Analysis of sequencing data and alpha diversity

**Fig. 2:**
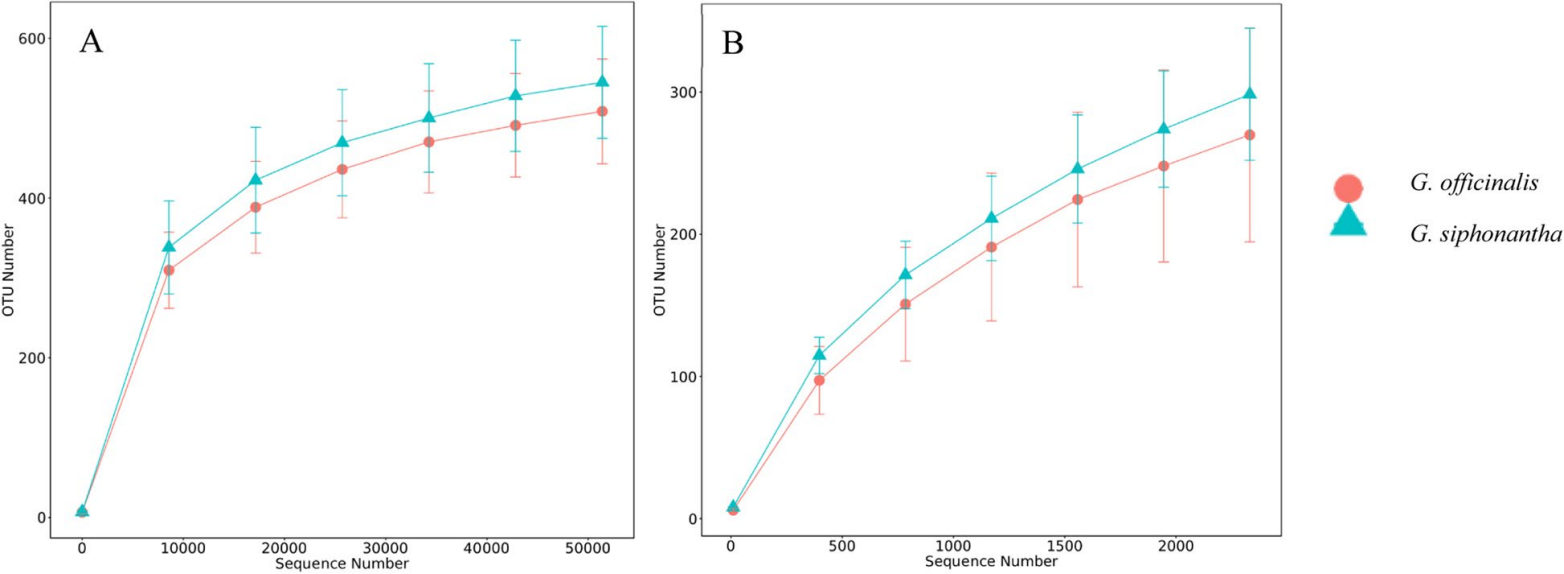
Rarefaction curves base on pyrosequencing of endophytic fungi (**a**) and bacteria (**b**) for each sample

A total of 130,792 and 121,736 effective tags were obtained for the fungal and bacterial samples (Table 1S), respectively. The goods_coverage of the all samples were higher than 0.961 (Table 2S), which indicates that the sequencing data can confidently reflect the community structure of endophytic fungi and bacteria for the all samples. The rarefaction curve can reflect the changes of species diversity and the richness with the sequencing amount. With the increasement of sequencing effort, the rarefaction curves of the samples tended to be stable based on the number of observed species, which indicated that the amount of sequencing data gradually tended to be reasonable (Fig. 2).

**Table 1:** Community diversity of endophytic fungi and bacteria of different *Gentiana* species

Sample	Endophytic fungi		Endophytic bacteria	
	shannon	chao1	shannon	chao1
*G. officinalis*	4.533 ± 0.795 b	547.886 ± 42.793 b	4.464 ± 0.757 b	405.438 ± 67.011 b
*G. siphonantha*	4.933 ± 0.692 a	608.698 ± 66.125 a	5.766 ± 0.188 a	556.387 ± 60.556 a

*Note*: Values are mean ± SD (*n* = 3). Different letters above the bars indicate the differences are significant at *p* < 0.05

**Table 2:** Metabolite content of *G. officinalis* and *G. siphonantha*

Sample	Gentiopicroside (mg/g)	Loganic acid (mg/g)	Swertiamarine (mg/g)	Sweroside (mg/g)
*G. officinalis*	129.92 ± 1.27 a	7.39 ± 0.09 a	2.52 ± 0.03 b	0.90 ± 0.02 b
*G. siphonantha*	131.22 ± 0.45 a	5.55 ± 0.03 b	2.62 ± 0.01 a	1.54 ± 0.01 a

*Note*: Values are mean ± SD (*n* = 3). Different letters above the bars indicate the differences are significant at *p* < 0.05

**Fig. 3:**
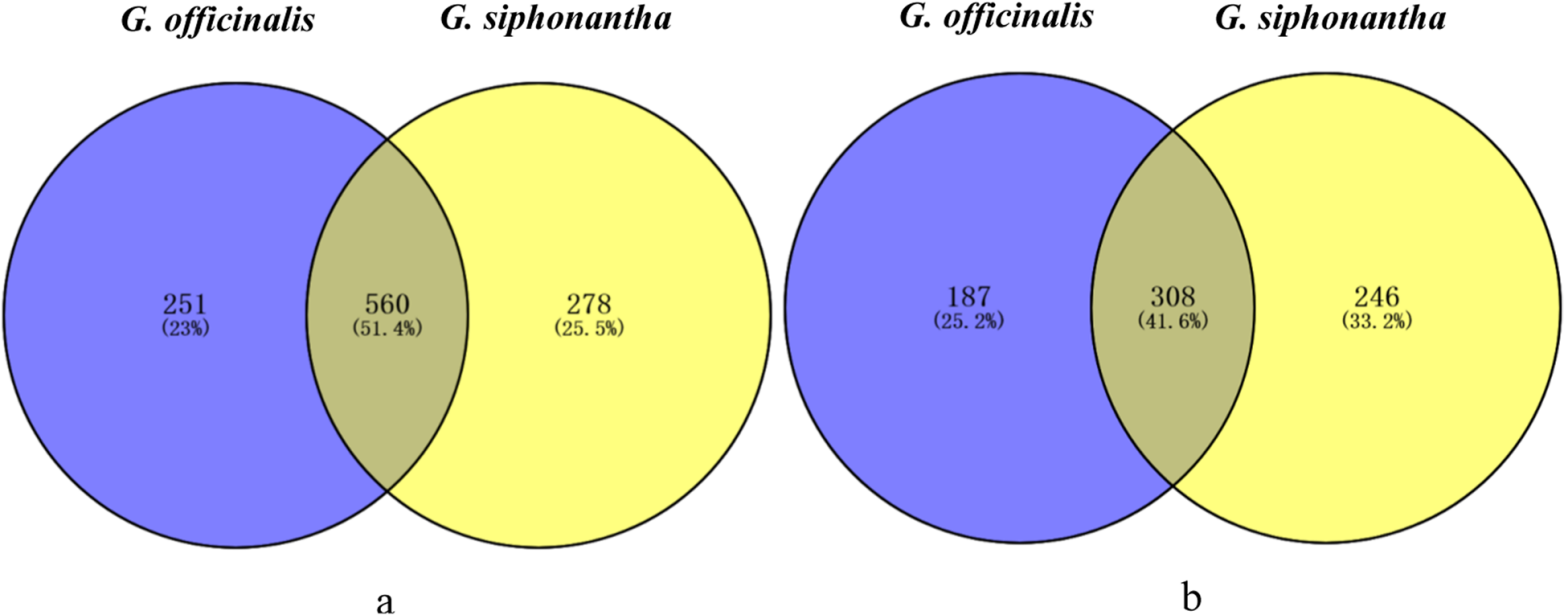
Venn diagram showing the fungal (**a**) and bacterial (**b**) OTUs shared among different *Gentiana* species

In all libraries, 278 fungal OTUs were exclusively recovered from *G. siphonantha*, 251 fungal OTUs were exclusively recovered from *G. officinalis* and 560 fungal OTUs were shared the both, while 246 bacteril OTUs were exclusively recovered from *G. siphonantha*, 187 bacterial OTUs were exclusively recovered from *G. officinalis* and 308 bcterial OTUs were shared the both (Fig. 3a, b).

Alpha diversity indices (Chao1 and Shannon’s diversity index) presented differences between *G. officinalis* and *G. siphonantha* samples. The fungal and bacteria communities’ richness and diversity of *G. siphonantha* was higher than *G. officinalis* (Table 1)*.*

### Community composition

**Fig. 4:**
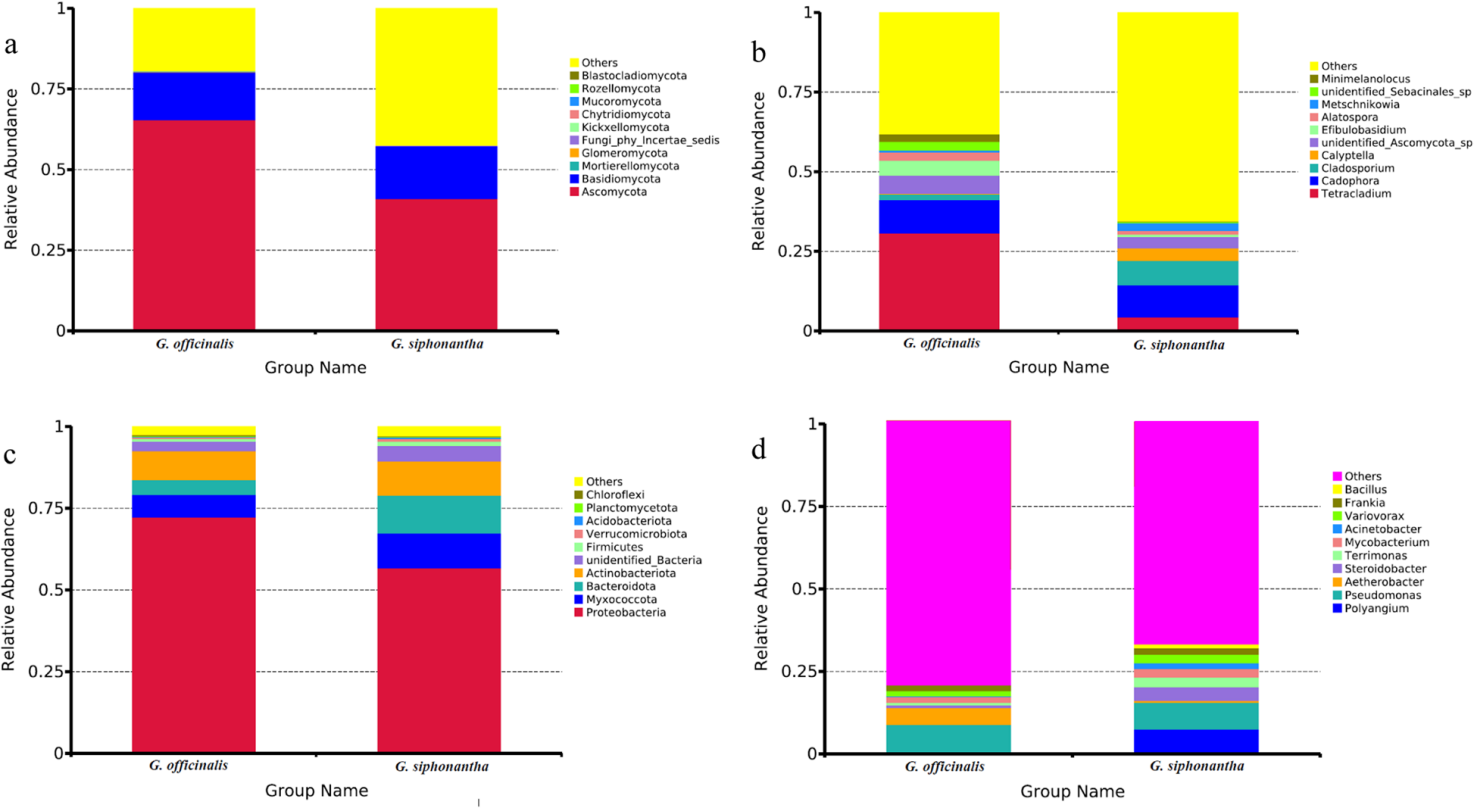
Relative abundances of the endophytic fungi at the phylum level (**a**), endophytic fungi at the genus level (**b**), endophytic bacteria at the phylum level (**c**), endophytic bacteria at the genus level (**d**) for each sample. Relative abundances are based on the proportional frequencies of the DNA sequences that could be classified. “Other” represents the total of relative abundance outside top ten maximum relative abundance levels

The fungal OTUs were assigned into 12 phyla and 285 genera. The dominant fungal phylum across all of samples was Ascomycota, with relative abundances ranging from 41.13 to 65.40% (Fig. 4a). At the genus level, *Tetracladium* was dominant genus in *G. officinalis* samples (30.87%), while *Cadophora* was dominant genus in *G. siphonantha* samples (9.93%) (Fig. 4b).

**Fig. 5:**
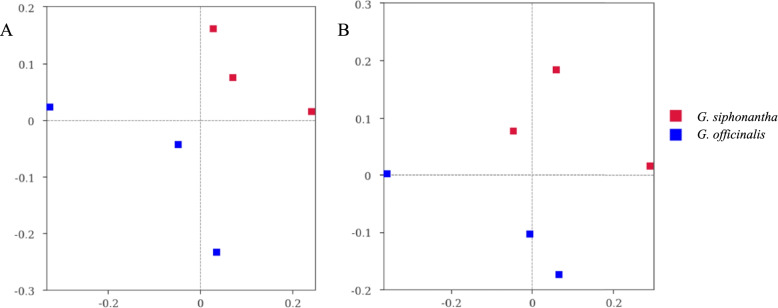
NMDS results of fungal (**A**) and bacterial (**B**) community composition. The digital number represented three biological replicates for each sample

The bacterial OTUs were assigned into 35 phyla and 296 genera. The dominant bacterial phylum across all of samples was Proteobacteria, with relative abundances ranging from 56.90 to72.39% (Fig. 4c). At the genus level, *Pseudomonas* was.

dominant genus in *G. officinalis* and *G. siphonantha* samples (8.29 and 8.05%) (Fig. 4d). As shown in Fig. 5, the community composition of fungal and bacterial endophyte varied among two *Gentiana* plants.

### Correlation analysis between endophytes and metabolites

**Fig. 6:**
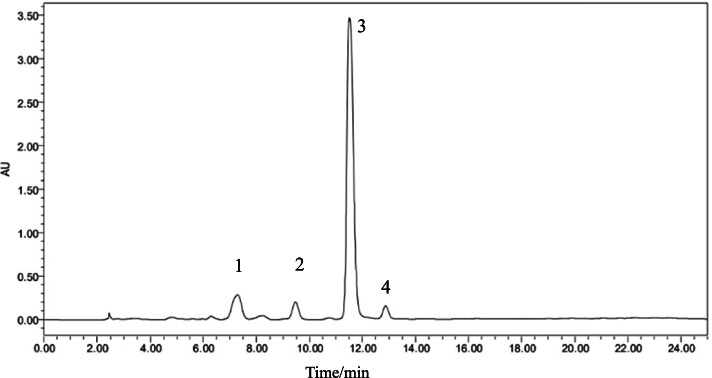
The HPLC of metabolite standards of *Gentiana* plants. Note: 1 is loganic acid, 2 is swertimarin, 3 is gentiopicroside, 4 is sweroside

Four secondary metabolites standards of *Gentiana* plants by HPLC as shown in Fig. 6, it indicated that five secondary metabolites of *Gentiana* plants can be effectively tested under the condition of this HPLC.

**Fig. 7:**
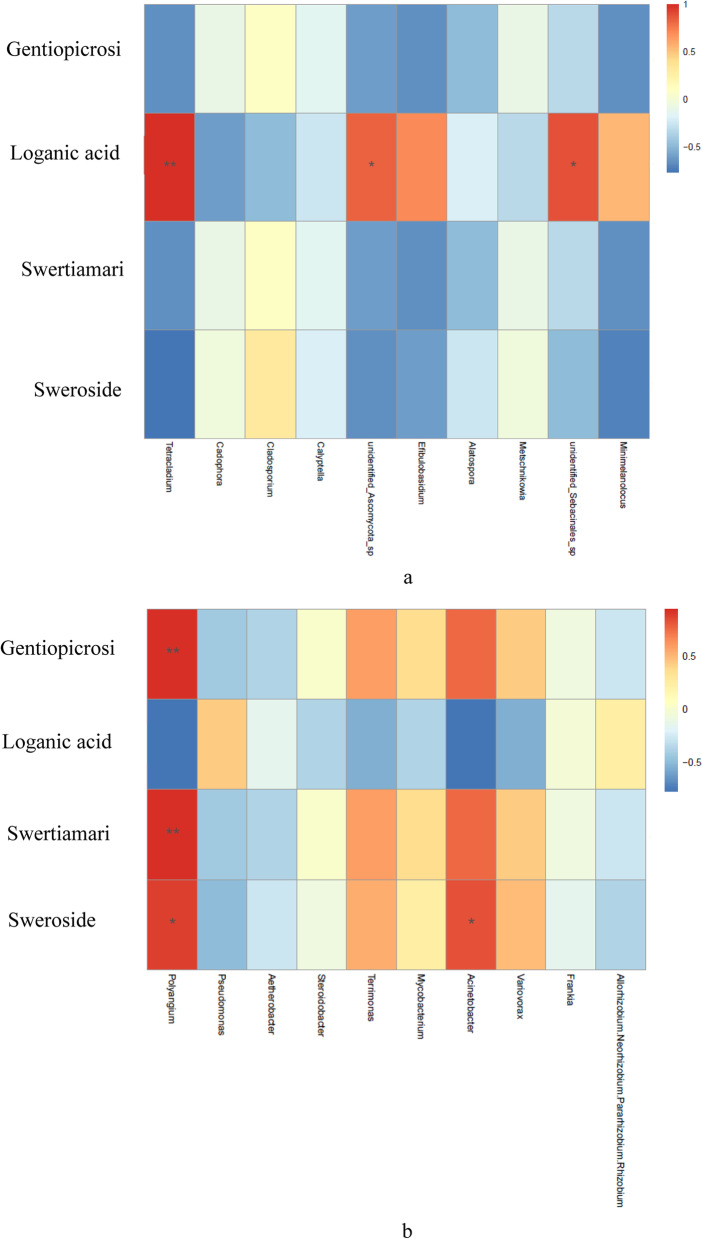
Correlation analysis between metabolites and top ten maximum relative abundance of endophytic fungi (**a**) and bacteria (**b**) at the genus level. Note: * indicate the differences are significant at *p* < 0.05, ** indicate the differences are significant at *p* < 0.01

As shown in Table 2, the four metabolites content of two species were difference. Among them, the gentiopicroside content of two species was no significant difference, while loganic acid, swertiamarine and sweroside content were significant difference (*P* < 0.05). Correlation analysis between metabolites and endophytes showed that *Tetracladium*, *unidentified_Ascomycota_*sp and *unidentified_Sebacinales_*sp were significantly positively correlated with the content of loganic acid (Fig. 7a). While *Polyangium* was significantly positively correlated with the content of gentiopicroside, swertiamarine and sweroside, *Acinetobacter* was only significantly positively correlated with the content of sweroside (Fig. 7b).

### PICRUST and FUNGuild functional prediction analysis

**Fig. 8:**
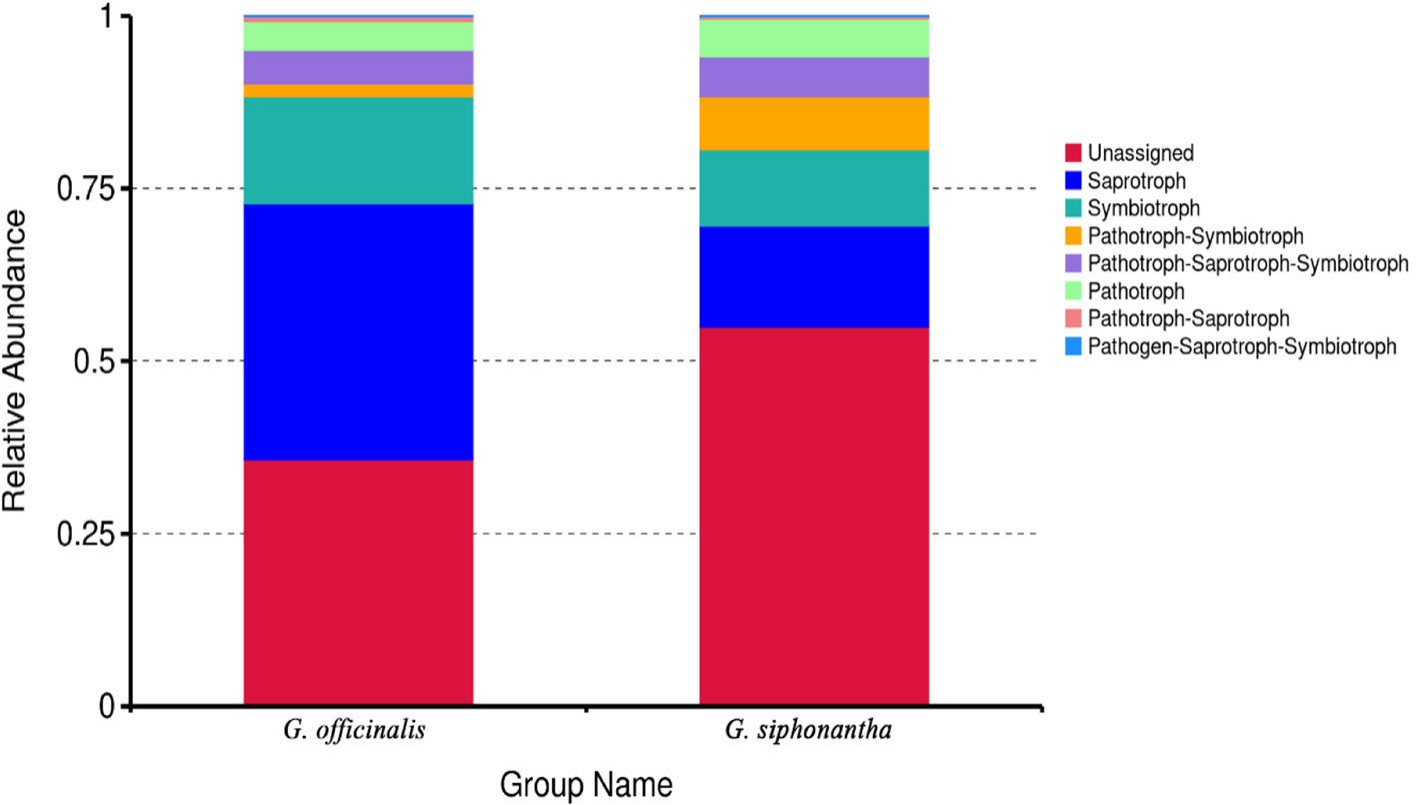
Relative abundance of predicted trophic mode of fungi

FUNGuild was used to predict the trophic modes of the fungal endophyte communities in the different samples. The results showed that seven trophic modes were classified, including Saprotroph, Symbiotroph, Pathotroph-Symbiotroph, Pathotroph-Saprotroph-Symbiotroph, Pathotroph, Pathotroph-Saprotroph, Pathogen-Saprotroph-Symbiotroph. Saprotroph was primary trophic mode of endophytic fungi in the two related species, the relative abundances were 14.66 to 37.00%, respectively (Fig. 8).

**Fig. 9:**
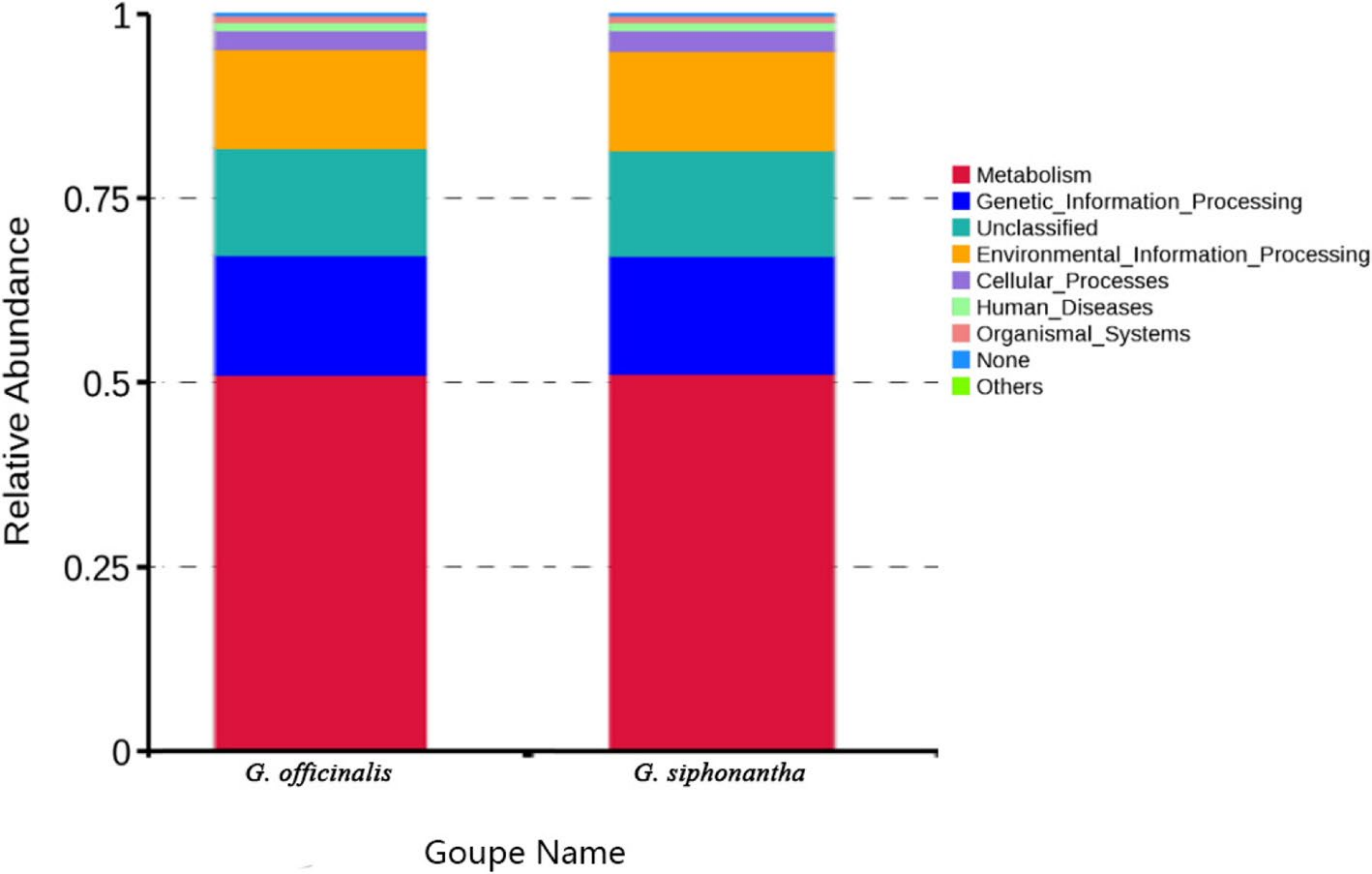
Relative abundance of predicted KEGG Orthologs functional profles (KEGG level 1) of bacteria

PICRUSt was used to predict the function of the bacterial endophyte communities in the different samples based on the KEGG (Kyoto Encyclopedia of Genes and Genomes) database, as shown in Fig. 9, metabolism pathway was the dominant function in two related samples, the relative abundances were 51.08 –51.20%, respectively (Fig. 9).

## Discussion

In this study, the dominant phyla of fungal and bacterial endophyte across all samples were ascomycota and proteobacteria, respectively (Fig. 1S). These two phyla were distributed in each sample, but their relative richness showed difference. Numerous studies have reported that the dominant phyla of fungal and bacterial endophytes were ascomycota and proteobacteria in many plants [22, 23]. At the genus level, dominant genera of fungal endophyte and their relative richness of two *Gentiana* samples existed difference, while dominant genera of endophytic bacteria was the same, these results were consistent with Soon et al. [24], who reported that dominant genera of fungal endophytes was different in the four *pinus* species. This result may be due to diversity of the endophytes was influenced by plant species, parts, and growth stage [6]. In this study, we only tested one sampling point and a single sampling time point, the observed information on endophyte diversity and community structure is limited. Therefore, the endophyte diversity of the two *Gentiana* plants under different sampling site and multiple time periods should be considered in the follow-up study.

Previous studies have reported that a lot of research focused that external ecological environment and the gene impact medicinal plants, such as temperature, rainfall, light and so on [14]. In our work, *G. officinalis* and *G. siphonantha* samples were collected from same production, and the both were mixed planting in the same field. We tested the metabolites content of two *Gentiana* species through HPLC, we found that the metabolites content of the two species were different. It indicated that the difference of metabolites content may be affected by internal factors, such as endophytes. Endophytes have a wide range of biosynthesis ability and can produce a variety of secondary metabolites with biological activities. Many studies have reported that endophytes can produce the same or similar substances as host secondary metabolites [25–27]. Correlation analysis between metabolites and endophytes showed that *Tetracladium*, *unidentified_Ascomycota_sp* and *unidentified_Sebacinales_sp* were significantly positively correlated with the content of loganic acid. While *Polyangium* was significantly positively correlated with the content of gentiopicroside, swertiamarine and sweroside, *Acinetobacter* was significantly positively correlated with the content of sweroside. It indicated that the content of loganic acid was correlated with endophytic fungi, the content of gentiopicroside, swertiamarine and sweroside were correlated with endophytic bacteria in the *G. officinalis* and *G. siphonantha*. However, Chen et al. [3] and Cui et al. [28] reported that that metabolites content of *Rheum palmatum* and *Cynomorium songaricum* were only correlated with endophytic fungi. Endophyte play a important role on the accumulation of secondary metabolite in medicinal plants [3], while endophyte may have different effects on the accumulation of secondary metabolites in different plants. This study preliminarily shows that there are abundant endophyte in the roots of two *Gentiana* plants, which are closely related to the content of secondary metabolites, which proves that endophyte may be involved in the accumulation of secondary metabolites of *Gentiana* plants, which is worthy of further study. In the next study, the isolation of endophyte from *Gentiana* plants and its inoculation into *Gentiana* plants to verify the effect and mechanism on the accumulation of secondary metabolites should be paid more attention.

PICRUSt can reliably predict the function of bacterial communities [29] and has been used to study bacterial functions of many plants [30]. We used PICRUSt to predict function of endophytic bacteria based on the high throughput sequencing results. The results showed that the metabolism was dominant function in two *Gentiana* plants. Those result was similar to the Pii et al. [31] study on the rhizosphere bacterial function of barley and tomato. Pepe-Ranney et al. [32] reported that endophyte originated from the rhizosphere microbiome, so the results were similar.

FUNGuild has been used to study the function of fungi, reflecting the specific functional classification of fungi. In recent years, it has been widely used in the study of fungal communities [33]. In this study, we used FUNGuild to predict fungal endophyte of *G. officinalis* and *G. siphonantha*. The results showed that saprotroph was dominant trophic mode in two related samples. Although FUNGuild was widely used, there were existing some limitations because of literature and data. Therefore, it is necessary to further study the classification and functional groups of soil fungi in order to comprehensively study the function of fungal endophyte.

## Supplementary Information

**Additional file 1: Table 1S.** The effective tags of endophytic fungi and bacteria of different *Gentiana* species. **Table 2S.** The goods_coverage of endophytic fungi and bacteria of different *Gentiana* species. **Figure 1S.** Relative abundances of the endophytic fungi at the phylum level (A) and endophytic bacteria at the phylum level (B). “Other” represents the total of relative abundance outside top ten maximum relative abundance levels.

## Data Availability

The 16S rRNA and ITS gene sequences of endophytes used in this manuscript have beensubmitted to the NCBI and the Accession number is SAMN21356694.
