# Supplementary material for: Analysis of endophyte diversity of two Gentiana plants species and the association with secondary metabolite
Source: BMC Microbiol. 2022 Apr 7;22:90. doi: 10.1186/s12866-022-02510-4 (PMC8988345; doi:10.1186/s12866-022-02510-4)
Supplement: Supplementary file 1 — Additional file 1: Table 1S. The effective tags of endophytic fungi and bacteria of different Gentiana species. Table 2S. The goods_coverage of endophytic fungi and bacteria of different Gentiana species. Figure 1S. Relative abundances of the endophytic fungi at the phylum level (A) and endophytic bacteria at the phylum level (B). “Other” represents the total of relative abundance outside top ten maximum relative abundance levels. [file 12866_2022_2510_MOESM1_ESM.docx]

Table 1S The effective tags of endophytic fungi and bacteria of different *Gentiana* species

| Sample | Endophytic fungi | Endophytic bacteria |
| --- | --- | --- |
| *G. officinalis* ① | 69,666 | 62,512 |
| *G. officinalis* ② | 66,282 | 58,118 |
| *G. officinalis* ③ | 61,546 | 69,428 |
| *Average* | 65,831 | 63,353 |
| *G. siphonantha* ① | 61,874 | 52,863 |
| *G. siphonantha* ② | 69,105 | 63,851 |
| *G. siphonantha* ③ | 63,903 | 58,434 |
| Average | 64,961 | 58,383 |

A total of effective tags = The average effective tags of *G. officinalis +* The average effective tags of *G. siphonantha*

Table 2S The goods_coverage of endophytic fungi and bacteria of different *Gentiana* species

| Sample | Endophytic fungi | Endophytic bacteria |
| --- | --- | --- |
| *G. officinalis* | 0.999 | 0.975 |
| *G. siphonantha* | 0.999 | 0.961 |


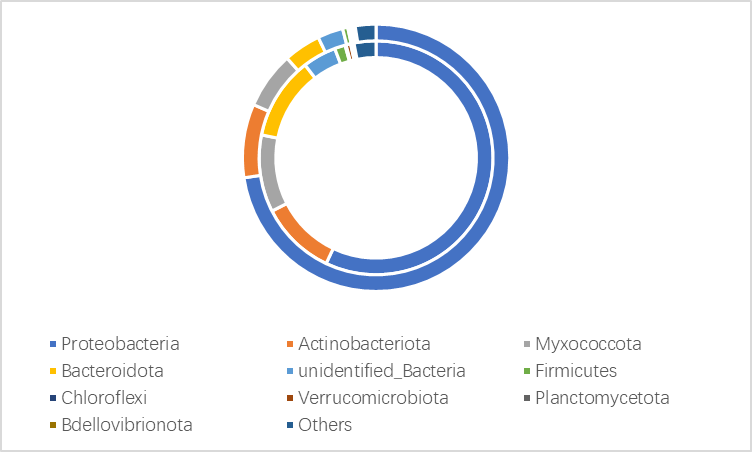


B

*G. siphonantha*

*G. officinalis*

Figure 1S. Relative abundances of the endophytic fungi at the phylum level (A) and endophytic bacteria at the phylum level (B). "Other" represents the total of relative abundance outside top ten maximum relative abundance levels.
